# *Beating the blues after Cancer*: randomised controlled trial of a tele-based psychological intervention for high distress patients and carers

**DOI:** 10.1186/1471-2407-9-189

**Published:** 2009-06-17

**Authors:** Suzanne K Chambers, Afaf Girgis, Stefano Occhipinti, Sandy Hutchison, Jane Turner, Rob Carter, Jeff Dunn

**Affiliations:** 1 Griffith Institute for Health and Medical Research, Griffith University, Brisbane, Australia; 2 Viertel Centre for Research in Cancer Control, Cancer Council Queensland, Brisbane, Australia; 3 Centre for Health Research & Psycho-oncology, Cancer Council NSW & University of Newcastle, Newcastle, Australia; 4 School of Medicine, University of Queensland, Brisbane, Australia; 5 Deakin Health Economics, Deakin University, Melbourne, Australia

## Abstract

**Background:**

The diagnosis and treatment of cancer is a major life stress such that approximately 35% of patients experience persistent clinically significant distress and carers often experience even higher distress than patients. This paper presents the design of a two arm randomised controlled trial with patients and carers who have elevated psychological distress comparing minimal contact self management vs. an individualised tele-based cognitive behavioural intervention.

**Methods/design:**

140 patients and 140 carers per condition (560 participants in total) will been recruited after being identified as high distress through caller screening at two community-based cancer helplines and randomised to 1) a single 30-minute telephone support and education session with a nurse counsellor with self management materials 2) a tele-based psychologist delivered five session individualised cognitive behavioural intervention. Session components will include stress reduction, problem-solving, cognitive challenging and enhancing relationship support and will be delivered weekly. Participants will be assessed at baseline and 3, 6 and 12 months after recruitment. Outcome measures include: anxiety and depression, cancer specific distress, unmet psychological supportive care needs, positive adjustment, overall Quality of life.

**Discussion:**

The study will provide recommendations about the efficacy and potential economic value of minimal contact self management vs. tele-based psychologist delivered cognitive behavioural intervention to facilitate better psychosocial adjustment and mental health for people with cancer and their carers.

**Trial Registration:**

ACTRN12609000301268.

## Background

### The mental health burden of cancer

It is estimated that from 2001 to 2011 the number of newly diagnosed cases of cancer will increase by 29% in women and 32% in men [1]. Cancer is the leading cause of burden of disease and injury in Australia, accounting for nearly one-fifth of the total disease burden [2]. The diagnosis and subsequent treatment of cancer is a major life stress that is followed by a range of well described psychological, social, physical and spiritual difficulties [3]. While over time most people diagnosed with cancer go on to adjust effectively to their changed life circumstances without clinical intervention, approximately 35% will experience persistent clinically significant distress such as anxiety and depression, adjustment disorders, fears about cancer recurrence, and post traumatic stress reactions, that for some will worsen over time [4,5]. As well, many partners of cancer patients report high levels of psychological distress, sometimes even greater than that of the patients [6,7]. The most powerful prospective predictor of longer term distress is current distress [8,9], hence there is a clinical imperative to identify patients and family members experiencing high distress and refer those people to accessible targeted psychosocial therapies [10].

A range of measures are available to screen for high psychosocial distress. Memorial Sloan Kettering Cancer Center, New York developed a simple single item Brief Distress Thermometer (BDT) and accompanying Problem Checklist [11] that has now been incorporated into clinical practice guidelines for psychosocial care after cancer and relevant consumer materials [12,13]. The BDT is free of charge; and easy to administer and interpret; and has comparable accuracy with longer distress screening instruments such as the 14 item Hospital Anxiety and Distress Scale and the 18 item Brief Symptom Inventory [14,15]. As such, the BDT is well suited for large-scale and repeated distress screening. There is also a well-established body of evidence demonstrating that psychosocial interventions increase wellbeing, improve adjustment and coping and reduce psychological distress in people affected by cancer [10,16]. A range of effective intervention approaches have been described using varying delivery formats, and these include cognitive behavioural therapy, relaxation techniques, psycho-education, supportive psychotherapy, and family and couples therapy. As well, in North America and Australia clinical practice guidelines for psychosocial care have been developed that provide recommendations for intervention [10,12,13,17]. However, despite the availability of guidelines and tools, evidence-based psychosocial care is the exception rather than the norm. Clinicians tend to overlook patients' psychosocial needs, often do not recognise depression and other psychiatric illnesses in their patients, and have limited response skills for managing patients' distress [8,18-21]. Routine screening for psychological distress is uncommon in acute treatment centres reducing the likelihood of detecting highly distressed patients and providing them with timely support [8]. Compounding this, while *some *psychosocial care is likely to be delivered to patients at diagnosis, it is uncommon at the time of treatment completion and will seldom include family members. For example, in a sample of 439 cancer patients treated at a tertiary cancer centre in Queensland, psychosocial care information was provided to only half of patients at diagnosis, and at the completion of treatment to less than one third [21]. Psychosocial care services that are responsive and accessible across the illness experience and beyond the acute treatment setting are urgently needed.

### Community-based approaches to psychosocial intervention

Increasingly, community based non-government organisations have taken on a role as providers of support services for cancer patients and their families. The most prominent and easily accessed of these are tele-based information and support services or helplines that are now available in the UK, Netherlands, Australia and North America [22-25]. Cancer helplines provide a potential assessment and referral point for patients and family members for psychosocial intervention both during and beyond their treatment experience within the acute health care setting. In Australia the Cancer Council Cancer Helpline is provided through a national toll free number (131120) and managed by each state Cancer Council. Telephone delivery provides a broad reach that addresses barriers to access such as geography, ill health, and cost. The Helpline service is staffed by nurses and allied health professionals who have experience and/or qualifications in oncology and who undergo additional training in psycho-oncology and decision support [26,27]. Helpline operators provide brief cancer information and emotional support typically in a single contact that includes an average of 8 minutes of verbal contact time followed by mailed written patient education materials. The service is underpinned by a constantly updated and extensive data base of community and hospital based services to facilitate referral across sectors. The use of a centralised call centre enables standardisation of procedures to be easily achieved with constant quality assurance, outcome monitoring and data collection built into the operational system. Policies and procedures to address issues such as confidentiality and duty of care are in place and are consistent with Australian Psychological Society professional standards and medicolegal obligations [28].

With regards to psychosocial intervention, population-based approaches need to address not only access, but also the costs of delivery that may impede dissemination. For example, individualised cognitive behaviour therapy has been found in meta-analyses to reduce anxiety and depression after cancer with follow up periods of up to eight months [29]. While these results are impressive, this approach will require specialist psychological staff, infrastructure to support delivery, and compliance by patients with routinised therapy sessions. In comparison, minimal contact approaches to psychological care with the use of self management materials for people with sub-threshold depression have been found to reduce the later incidence of major depression in patients in primary care [30]. Efficacious self management has been defined as where individuals are able to monitor their own condition and undertake the necessary cognitive, behavioural and emotional responses to effect a satisfactory quality of life [31]. Self management interventions have been shown to have consistent positive effects on mood across a range of chronic illnesses [31] and so have great potential as a cost effective method of providing psychological support to people affected by cancer. Proposed advantages of self management approaches are equity, accessibility and choice [32]. However, it is not yet clear for which distressed patients a minimal contact approach would be efficacious, and for whom more in depth individualised approaches are necessary. For example, it may be that patients and carers with borderline distress will return to pre-morbid functioning with minimal contact approaches while clinically distressed people will require more in depth care to achieve similar benefits. This is a research question that is of critical importance in order to guide the cost-effective delivery of psychosocial oncology care services for the population.

For practical capacity for immediate translation into the community, psychosocial distress screening and intervention should be nested within an available service infrastructure. The Cancer Helpline provides a unique service infrastructure with demonstrated feasibility for the delivery of well-evaluated tools to diagnose psychosocial distress and depression/anxiety in people with cancer in our community, and their family members and carers; and potential for the delivery of psychosocial interventions. The Cancer Helpline has unique broad access intervention capacity. The service has national coverage with uniform service standards; is promoted in national patient and clinician educational materials as a contact for evidence-based cancer support and information; national media coverage about cancer consistently uses the Cancer Helpline as a referral point for the public; the use of a tele-based delivery mode with a toll free contact number addresses geographical, disability, illness and cost barriers to access; the service is community based and accessible at any point in the illness continuum. Each year nationally over 30,000 patients and carers contact the Helpline. However, research is needed to extend the Helpline to include in depth psychosocial intervention; and to assess the relative efficacy of minimal contact approaches with individualised care. The present study will evaluate the relative efficacy and cost-effectiveness of these two approaches to psychosocial intervention for people distressed by cancer that are suitable for immediate population-based translation.

## Methods/design

### Study aims and hypotheses

The overall aim of the proposed study is to compare the efficacy and cost effectiveness of minimal contact self management materials vs. a tele-based psychologist delivered cognitive behavioural intervention in improving psychological outcomes for high distress cancer patients and carers over a 12 month period. Callers who contact The Cancer Council Cancer Helpline in Queensland and New South Wales over a five month time period will be screened for psychosocial distress and those callers with *elevated distress *will be randomised to one of the two study arms. The two study arms will comprise: (1) minimal contact self management vs. (2) five sessions of an individualised tele-based cognitive behavioural intervention delivered by a clinical psychologist.

Study hypotheses: It is hypothesised that 3, 6, and 12 months after recruitment:

1. Participants in both study arms will experience significant improvements in mental health compared to baseline levels including: reduced anxiety and depression; cancer specific distress; unmet psychological supportive care needs; and increased positive adjustment and quality of life.

2. Participants with *borderline anxiety/depression *who receive the minimal contact self management condition will experience significantly less anxiety and depression; less cancer specific distress, lower unmet psychological supportive care needs, higher positive adjustment and improved quality of life **by comparison **to participants with *high anxiety/depression *who receive the minimal contact self management condition.

3. Participants with *high anxiety/depression *who receive the individualised tele-based cognitive behavioural intervention condition will experience significantly less anxiety and depression; less cancer specific distress, lower unmet psychological supportive care needs, higher positive adjustment and improved quality of life **by comparison **to participants with *high anxiety/depression *who receive the minimal contact self management condition.

4. That from a health sector perspective, the individualised tele-based cognitive behavioural intervention is more 'cost-effective' compared to the minimal contact self management comparator for *high anxiety/depression participants*, **and **minimal contact self management is more cost-effective than the individualised tele-based cognitive behavioural intervention for *borderline anxiety/depression participants*; where $50,000 per QALY is taken as the benchmark for cost-effectiveness in Australia.

### Intervention

The 'self help' self management materials will consist of an initial single 30-minute telephone support and education session with a nurse counsellor who will provide feedback to the participant about his/her levels of distress and instruction in evidence based strategies to improve adjustment. A self management manual will be developed purposefully for this study that will include self help in mood/stress management skills; problem solving approaches to cancer-related concerns; patient education about a healthy lifestyle to promote wellness and optimise quality of life; strategies for mobilising personal and community support networks to reduce isolation and seek sustainable social support. For the individualised cognitive behavioural intervention (therapist delivered), five weekly one-hour sessions of tele-based counselling from a clinical psychologist will be delivered that include psychoeducation, skills in stress reduction, problem-solving, cognitive challenging and enhancing relationship support. Cognitive behavioural approaches are well established with regards to their clinical efficacy for anxiety and depression in cancer and individualised approaches are more effective than groups [29]. The individualised counselling sessions will follow principles of cognitive-behavioural therapy and will utilise an adult learning approach in which participants self-select goals to focus on during the intervention. Content will include assigned behavioural homework that will be supported by work and tip sheets. Components that target challenges associated with cancer treatments (e.g., pain, sleep disturbance, fatigue) will be additionally selected if relevant. Minutes of counselling time will be recorded for inclusion in analyses for both arms.

### Participants

Study inclusion criteria will be that the patient/carer participants must: (1) be a cancer patient or carer ≥ 18 years of age who has called the Cancer Helpline for information or support (2) have a score of 4 or more on the Brief Distress Thermometer at the time of the Helpline contact (3) be able to read and speak English (4) have no previous history of head injury, dementia or psychiatric illness.

Callers who meet the selection criteria will be offered entry into the study by the Helpline operator at the time of the call; and once verbal permission to be contacted is obtained will be posted written consent and information forms. To minimise the period of time between calling the Helpline and randomisation into a support arm of the study, verbal consent to take part in the study will be obtained by project staff and audio taped prior to written consent. Baseline assessment will be conducted via telephone interview to prevent potential delays in receiving support. Participants will then be randomly allocated to receive either self management or the five session cognitive behavioural intervention. In all, 140 patients and 140 carers per condition will be recruited (allowing for a total of 280 people per treatment arm; 560 participants in total). On the basis of simulation work conducted by Raudenbush and Xiao-Feng [33], the present study, with 4 measurement periods and a total N of 560 will have power of at least .90 to detect a moderate longitudinal effect size. This sample size will allow for identification and investigation of subgroups of patients and carers (high vs. low/borderline distress) on the basis of their response to psychological intervention.

### Study Integrity

Ethical approval has been obtained from the Griffith University Human research ethics Committee. The study design will be guided by the CONSORT statement [34]. Randomisation to study condition will occur following the completion of baseline assessment. Assessments will be by self-report pen and paper measures and project staff tracking assessments will be blinded to condition. Randomisation will be stratified by patient/carer and state (QLD vs NSW) and will occur in blocks of 10, with each condition randomly generated 5 times within each block to ensure an unpredictable allocation sequence with equal numbers of participants in each group at the completion of each block. This sequence will be undertaken by the project manager and concealed from investigators. Therapy will be manualised and all intervention calls audiotaped and reviewed to monitor treatment adherence. All analyses will be conducted on the basis of intention to treat.

### Measures

A computer assisted telephone interview will be undertaken for the baseline assessment; subsequent assessments will be administered by mail at 3, 6 and to 12 months after recruitment. A short telephone interview at follow-up time points will also re-assess participants' use of support services. Primary outcomes are anxiety and depression, cancer specific distress and unmet psychological supportive care needs. Secondary outcomes are positive adjustment and overall quality of life.

Background variables will include socio-demographic and disease variables (e.g. cancer site and stage, medical treatments received, use of alternative therapies) and previous (past month) and current use of support services.

#### Distress Screening

The single item Brief Distress Thermometer (BDT) will be used to screen for global psychological distress [13]. This scale has good sensitivity and specificity when used with a cut off point of ≥ 4 [14].

#### Psychological Distress

The Brief Symptom Inventory – 18 (BSI-18) will assess psychological distress through three subscales of anxiety, depression, and somatisation [35].

#### Cancer Specific Distress

The Impact of Events Scale (IES: [36,37]) will be used to assess cancer specific distress. The IES has two subscales that measure the extent to which participants are experiencing intrusive thoughts about cancer and avoiding thinking about cancer [38].

#### Unmet Psychological Supportive Care Needs

The Supportive Care Needs Survey Short Form 34 (SCNS-SF34) will assess cancer patients' need for help over the last month across 5 domains: psychological, health systems and information, patient care and support, physical and daily living, and sexuality needs [39,40]. The Supportive Care Needs Survey for Partners and Caregivers (SCNS-P&C45) will assess carers' need for help over the last month.

#### Positive Adjustment

Positive adjustment will be measured with a 21-item Posttraumatic Growth Inventory (PTGI) assessing perceived positive life changes occurring after a diagnosis of cancer [41]. Domains assessed include strengthened relationships with others, appreciation of life, personal strength, new priorities, spiritual/religious growth.

#### Quality of Life

Health related quality of life will be measured with the Assessment of Quality of Life – 2 (AQoL-2), which is a more recent version of the original 15-item AQol. The AQoL-2 is a 20-item scale assessing quality of life on six dimensions; including independent living, social, mental health, coping, pain, and sensory perception [42]. The AQoL will also be used to assess the cost-effectiveness of the intervention options. AQoL utility data analysis will be used to estimate Quality-adjusted life years (QALYs).

### Statistical Analyses

The study is a multivariate, two condition randomized controlled trial with repeated measures across time. The analytic approach will be a multilevel model (MLM) in which measurement occasions (level 1) are nested within persons (level 2) and in which state/program differences are represented as a fixed effect at level 2 whose interaction with time represents differential adjustment and distress trajectories for the two groups. A key advantage of this approach is flexibility in dealing with missing data owing either to random or non-random attrition. Further, subgroups of patients and carers who respond differentially to the program will be identified using growth mixture modelling that is a related longitudinal procedure that identifies cases with similar distress and adjustment trajectories across time [43]. Jo's [44] results suggest that the proposed sample size will be sufficient to examine subgroups of responders.

### Economic Analyses

A trial-based economic evaluation will be conducted that integrates the efficacy and quality of life outcomes data with comparative cost data on the intervention options. The various distress indicators and the unmet needs data will be built in incremental cost-effectiveness analysis (CEA), while the quality of life data will be used in cost-utility analysis (CUA) using the Brazier algorithm. The feasibility of adding a modelled economic evaluation (to broaden the target population; lengthen the time horizon beyond that of the trial; and estimate cost offsets) will also be carefully assessed, having regard to trial results and the strength of evidence in the literature to underpin key modelling assumptions.

Costs and outcomes will be assessed from a health sector perspective, but with a focus on 'government as 3^rd ^party funder'. Cost impacts that fall on patients and their carers/families will be reported to the extent they can be assessed from the activity data; but this collection will not be complete. Pathway analysis and patient flowcharts (incorporating decision tree analysis) will be used to clearly identify and cost the activity components for each arm of the trial. Standard discounting will be applied to both costs and outcomes, together with detailed sensitivity testing (using @RISK probabilistic software).

## Discussion

This study will address a critical research question: what is the efficacy and cost-effectiveness of population based strategies for promoting optimal psychosocial adjustment and mental health for people with cancer and their carers who are at risk? To date, intervention studies have typically not been adequately powered to look differentially at effects for high versus borderline/low distress patients and carers; or included economic analyses for cost-benefit. This research will overcome these limitations. Most importantly however, the proposed study will also provide a strategy for delivering in depth intervention to high distress individuals that can be immediately translatable into broad reach tele-health services both in Australia and internationally; and potentially replicated for other chronic illnesses.

## Abbreviations

BDT: Brief Distress Thermometer; QALY: Quality-adjusted life years; HADS: Hospital Anxiety and Depression Scale; QLD: Queensland; NSW: New South Wales; BSI-18: Brief Symptom Inventory 18; IES: Impact of Events Scale; SCNS-SF34: Supportive Care Needs Survey Short Form 34; SCNS-P&C45: Supportive Care Needs Survey for Partners and Caregivers 45; PTGI: Posttraumatic Growth Inventory; AQol: Assessment of Quality of Life; MLM: Multilevel model; CEA: Cost effectiveness analysis; CUA: Cost utility analysis.

## Competing interests

The authors declare that they have no competing interests.

## Authors' contributions

SKC and JD developed the study concept, aims and initiated the project. AG, SO, SH, JT and RC assisted in the further in depth development of the protocol. SKC was responsible for the drafting of the manuscript. SKS, AG, SO, SH, JT will implement the protocol and oversee collection of the data. All authors have read and approved the final manuscript.

## Pre-publication history

The pre-publication history for this paper can be accessed here:

http://www.biomedcentral.com/1471-2407/9/189/prepub
